# The Assessment of Serum Cytokines in Oral Squamous Cell Carcinoma Patients: An Observational Prospective Controlled Study

**DOI:** 10.3390/jcm11185398

**Published:** 2022-09-14

**Authors:** Ana Caruntu, Cristian Scheau, Elena Codrici, Ionela Daniela Popescu, Bogdan Calenic, Constantin Caruntu, Cristiana Tanase

**Affiliations:** 1 Department of Oral and Maxillofacial Surgery, “Carol Davila” Central Military Emergency Hospital, 010825 Bucharest, Romania; 2 Department of Oral and Maxillofacial Surgery, Faculty of Dental Medicine, “Titu Maiorescu” University, 031593 Bucharest, Romania; 3 Department of Physiology, Carol Davila University of Medicine and Pharmacy, 050474 Bucharest, Romania; 4 Biochemistry-Proteomic Laboratory, Victor Babes National Institute of Pathology, 050096 Bucharest, Romania; 5 Centre for Immunogenetics and Virology, Fundeni Clinical Institute, Carol Davila University of Medicine and Pharmacy, 258 Fundeni Road, 022328 Bucharest, Romania; 6 Department of Dermatology, Prof. N.C. Paulescu National Institute of Diabetes, Nutrition and Metabolic Diseases, 011233 Bucharest, Romania; 7 Department of Molecular Biology, Faculty of Medicine, “Titu Maiorescu” University, 031593 Bucharest, Romania

**Keywords:** oral squamous cell carcinoma, cytokines, inflammation, carcinogenesis, tumor microenvironment

## Abstract

Background: The oral squamous cell carcinoma (OSCC) tumor microenvironment (TME) is a complex interweb of cells and mediators balancing carcinogenesis, inflammation, and the immune response. However, cytokines are not only secreted within the TME but also released by a variety of other cells that do not comprise the TME; therefore, a thorough assessment of humoral changes in OSCC should include the measurement of serum cytokines. Methods: We assessed the role of various serum cytokines in the evolution of OSCC, before and after treatment, versus a control group. We measured the serum concentrations of MIP-1α, IL-1β, IL-4, IL-6, IL-8, IL-10, and TNF-α. Results: Significantly higher values (*p* < 0.01) were noted for IL-1β, IL-6, IL-8, IL-10, and TNF-α in the OSCC group before treatment (n = 13) compared with the control group (n = 14), and the increased concentrations persisted after treatment (n = 11). Furthermore, the variations in the values of MIP-1α, IL-1β, IL-10, and TNF-α are correlated both before and after treatment (*p* < 0.01). In the pretherapeutic group, IL-6 and IL-8 concentrations also correlate with IL-1β and IL-10 serum levels (*p* < 0.01), while in the posttherapeutic group, IL-4 varies with MIP-1α and TNF-α (*p* < 0.01). Conclusion: In OSCC patients, serum cytokine levels are significantly higher compared with control, but they are not significantly altered by treatment, therefore implying that they are also influenced by systemic factors. The interactions between all involved cytokines and the various pathways they regulate warrant further studies to clarify their definitive roles.

## 1. Introduction

Head and neck squamous cell carcinoma (HNSCC), a heterogeneous group of malignancies arising from the upper aerodigestive mucosa, is the most frequent cancer of the craniocervical region and the sixth most common malignancy worldwide, with an expected increase in incidence in the following years [1]. Significant efforts have been made to develop new methods for the prevention and early diagnosis of HNSCC [2,3]. Among these, we mention salivary metabolomics, an approach that entails the assessment of various metabolites in saliva with the intent of detecting metabolic changes in patients with HNSCC [4,5,6].

Oral squamous cell carcinoma (OSCC) is the main subtype of head and neck cancer, and there are strong data suggesting an interplay between carcinogenesis, inflammation, and impairment of the immune system in this malignancy [1,7]. In a recent study that evaluated the cellular component of OSCC-associated immune–inflammatory response, our research group emphasized profound and persistent alterations of peripheral blood lymphocyte subtypes in OSCC patients compared with control subjects [8]. In order to expand the knowledge regarding the impact of OSCC on the systemic immune–inflammatory response, our group has turned its interest to the investigation of specific cytokines that sustain the molecular cross-talk between immune and non-immune cells.

Cytokines are not only released by circulating immune cells but also by various cells in the tumor microenvironment and by the tumor cells themselves [9,10]. Their serum levels are influenced not only by tumor progression but also by the treatment, the response to tumor evolution, and potentially other confounding variables, such as the structure of the extracellular matrix and matrix metalloproteinases [11,12]. Thus, the study of cytokines is likely to provide insights into these complex interconnections. Recent research shows that cytokines may be involved in HNSCC tumorigenesis, particularly involving oral mucosa, and therefore might play diagnostic and therapeutic roles [13]. Pro-inflammatory cytokines such as IL-1α and IL-1β are found within the HNSCC tumor microenvironment and have been linked to tumor development and spread [14]. The expression of IL-1α was found to be a prognostic factor for the development of distant metastases in patients with HNSCC [15]. A recent study has shown the potential of IL-1α as an enhancer of the immune response in EGFR-positive HNSCC [16]. Additionally, an increased expression of IL-1β was correlated with longer survival of HNSCC patients treated with cetuximab [17]. Other cytokines such as the pro-inflammatory interleukins IL-6 and IL-8 and the anti-inflammatory cytokines IL-4 and IL-10 were also implicated in the immune response to HNSCC and considered biomarkers in this group of diseases [18,19,20].

Taken together, these data suggest that cytokines may be used as indicators for OSCC development but also as biomarkers, prognostic predictors, and potential therapeutic targets. Therefore, establishing a cytokine profile could be instrumental in patient management.

This observational prospective controlled study aimed to detect differences between OSCC patients and matched healthy controls in serum levels of cytokines before treatment and three months after. We advanced the hypothesis that OSCC patients would present changes in their cytokine levels after treatment and that these levels might be different from normal values (i.e., compared with a control group) due to other involved factors such as the modulation of the immune response. The present paper is a continuation of our previous work with the intent of highlighting the role of the humoral immune component involved in the evolution of OSCC.

## 2. Materials and Methods

### 2.1. Patient Enrollment

Our research is an observational study that included patients with OSCC and healthy controls. The study subjects were enrolled for a period of 6 months between April 2019 and November 2019. The study was conducted in accordance with the Declaration of Helsinki (1964) with the approval of the Local Ethics Committee (333/28.03.2019). The patients were recruited from the Department of Oral and Maxillofacial Surgery of the Carol Davila Central Military Emergency Hospital in Bucharest and respected the following inclusion criteria:

- Diagnosed with OSCC, confirmed by histopathology;
- Primary tumor sites were tongue, oral mucosa, and labial lesions;
- Patient eligible for curative surgery according to disease staging;
- No prior history of malignancies with or without treatment;
- No other concurrent cancers, severe disorders, or immune system pathology (such as decompensated systemic or metabolic diseases, or autoimmune diseases).

The control group was chosen to include subjects who were age-matched and gender-matched, and the inclusion criteria were: no prior malignancies, no inflammatory or infectious pathology, and no medication in the recent history. In addition, all subjects included in the control group denied tobacco and alcohol consumption.

### 2.2. Investigations

#### 2.2.1. Clinical Examination and Imaging Studies

The patients in the study group followed a specific protocol that included a thorough clinical examination (personal and disease history, inspection, and palpation), imaging studies (head, neck, and thoracic contrast-enhanced CT/MRI scans) to determine the clinical staging of the disease, drawing venous blood for routine blood work, measuring multiple biochemical and hematological parameters, and evaluating serum cytokine levels. The OSCC patients were then submitted to surgical treatment that involved tumor excision with appropriate safety margins, appropriate defect reconstruction, and lymph node dissection in selected cases. Post-surgically, patients underwent radiotherapy and chemotherapy in accordance with the treatment plan indicated by the oncology board of the hospital center. A follow-up was scheduled for 3 months after the completion of treatment when another assessment of the serum cytokine levels was performed, an assessment that was attended by 11 of the patients. Two patients dropped out of the study and did not return for the follow-up visit due to personal reasons (relocation for one patient and motility disability in an elderly patient).

#### 2.2.2. Analysis of Serum Cytokines

The assessment of cytokines was performed with xMAP Array analysis. The xMAP array was performed according to the manufacturer’s protocols and analyzed using a Luminex^®^ 200™ system (Luminex, Luminex Corp., Austin, TX, USA). Serum cytokine levels were determined using the MILLIPLEX MAP Human Cytokine/Chemokine Magnetic Bead Panel Kit (Merck-Millipore, Billerica, MA, USA), with the following analyte-specific bead sets: MIP-1α, IL-1β, IL-4, IL-6, IL-8, IL-10, and TNF-α. Briefly, in a 96-well plate, the beads were incubated with the samples, controls, and standards overnight at 4 °C. All further incubations with the detection antibodies and streptavidin phycoerythrin (SAPE) conjugate were performed at room temperature in the dark with shaking at 800 rpm. Data acquisition and analysis were performed using xPONENT 4.2 software (Luminex, Luminex Corp., Austin, TX, USA); the calibration curves were generated with a 5-parameter logistic fit.

### 2.3. Statistical Analysis

The data were collected and analyzed using MedCalc^®^ Version 14.8.1 (MedCalc Software bvba, Ostend, Belgium) and SPSS software version 23 (IBM, Armonk, NY, USA). The distribution of the data was tested for normality using the Shapiro–Wilk test. The chi-squared test was used to analyze the significance of the gender differences between groups, and the t-test was used for the comparison of ages between groups. In order to compare the levels of cytokines among the three study groups, the Kruskal–Wallis test with post hoc pairwise comparisons was used. The data did not carry a normal distribution, and therefore, this nonparametric test was considered. Pearson’s correlation coefficient was calculated for assessing the degree of association between cytokines. Results are presented as mean ± standard error (SE) or standard deviation (SD) where appropriate. In the case of nonparametric tests, the results are presented in the form of medians and quartile ranges. Statistical significance was considered at *p* values < 0.05.

## 3. Results

### 3.1. Patient Demographics

The characteristics of the subjects included in the patient and control groups are presented in Table 1 and Table 2. As expected, we found no statistically significant differences between the study and control groups in regard to gender or age.

In our group of patients, the male:female ratio was 3:10. More than half of the patients were smokers (54%), while alcohol consumption was confirmed in 5 patients (38%). The primary sites of the tumors diagnosed in our group of patients were the tongue, floor of the mouth, retromolar region, lower lip, buccal area, and gingiva. The TNM staging was I in 2 patients (15%), II in 5 patients (38%), III in 1 patient (8%) and IVA in 5 patients (38%).

### 3.2. Comparative Analysis of Serum Cytokine Levels between Study Groups

The comparative analysis of the serum cytokines levels showed significant differences between the OSCC group and the control group for all cytokines except MIP-1α and IL-4. No significant differences in serum cytokines expression were detected in the OSCC group between before treatment and at the follow-up visit (Table 3). All analyzed parameters revealed similar patterns of changes between groups. We found significantly higher levels of IL-1β, IL-6, IL-8, IL-10, and TNF-α in OSCC patients, in both pre- and posttherapeutic determinations, compared with healthy controls. Furthermore, in the pretreatment OSCC group, the mean IL-6 was 40 times higher, IL-8 was 17 times higher, and TNF-α was 8 times higher, while for the rest of the cytokines—IL-1β and IL-10—the mean values were 6 times higher than the control group (Table 3, Figure S1).

### 3.3. Correlation Analysis between Serum Cytokines in OSCC Patients

An in-depth analysis of the cytokine profile in the OSCC group revealed significant correlations between many cytokines and chemokines, within both the pretherapeutic and posttherapeutic settings (Table 4 and Table 5). No significant correlations in serum cytokines levels were detected in the comparison between pre- and posttreatment values. In the pretreatment determination, IL-8 was significantly correlated with all the other cytokines assessed in our study. However, after treatment, the correlations were maintained only with IL-10 and IL-1β. Furthermore, MIP-1α revealed significant correlations with IL-10, IL-1β, and TNF-α, in both pre- and posttreatment determinations. In addition, IL-4 was significantly correlated with MIP-1α after treatment initiation. Along with IL-8 and MIP-1α, IL-10 revealed significant correlations with IL-1β, IL-4, and IL-6 but only in treatment-naive patients. After treatment, these correlations did not maintain their statistical significance. In addition, in the pretreatment determination, IL-6 also significantly correlated with IL-1β, while in the post-treatment group, IL-4 correlated significantly with both TNF-α and MIP-1α.

## 4. Discussion

The results of our study identify increased serum levels of several cytokines in OSCC patients. Their concentrations decrease insignificantly after treatment and do not achieve the normal levels identified in healthy controls. Moreover, increased values of certain cytokines are correlated in patients before and after treatment, suggesting their interrelation and common roles.

Cytokines are essential, ubiquitary molecules with multiple origins and a variety of effects in normal and pathological conditions [21]. Cytokines are produced not only by immune cells but also by the tumor microenvironment, as well as by virtually any nucleated cell [9]. The roles of cytokines in HNSCC, particularly involving oral mucosa, are multiple, and they are often antagonists between each other; their involvement in the tumor development, progression, invasion, and chemoresistance was demonstrated in multiple studies and is the reason why the scientific focus has extended from the tumor cells to their messengers [10,11]. In our previous work, we described the changes in the peripheral blood lymphocyte subtypes in OSCC patients [8]; however, it is clear that a more complex and accurate vision of the connections between the immune response and carcinogenesis should also include the evaluation of cytokines.

Our study tested a variety of serum cytokines and chemokines that have been identified in the tumor microenvironment of HNSCCs and were recognized as potentially playing a role in cancer development and dissemination [22,23,24,25,26]. These molecules have complex roles and intricate effects on inflammation, immunity, and tumoral metabolism.

We have shown that the levels of ILs 1β, 6, 8, 10, as well as TNF-α, are higher in treatment-naive patients with OSCC compared with healthy controls. These findings are supported by previous reports. Chen et al. [27] reported increased IL-1α, IL-6, and IL-8 levels in the sera of HNSCC patients, findings later confirmed by St. John et al., who reported elevated IL-6 and IL-8 in the serum and saliva, respectively, in patients with OSCC [28]. IL-10 was previously identified in higher concentrations in HNSCCs in various studies [19,29], and a direct correlation between overall values and tumor stage was also reported [30,31,32].

Another relevant finding of our study was that cytokine levels maintain their increased values after treatment when compared with control subjects, suggesting the stability of changes in the immune–inflammatory response and the extent of their systemic impact. Our results are supported by previous research. A longitudinal study performed by Mytilineos et al. showed that MIP-1α, IL-1, IL-4, IL-6, IL-10, and TNF-α levels do not change significantly after treatment when compared with pretherapeutic values [33].

Furthermore, we showed that serum cytokine concentrations are correlated in both the pretherapeutic and posttherapeutic study groups. This might be explained by their specific roles, common activators, interdependency, and interdetermination [34].

MIP-1α is a pro-inflammatory chemokine released by a variety of cells, including monocytes and macrophages, and it has important chemotactic abilities that have been revealed in inflammations as well as various cancers [35]. In consequence, MIP-1α was considered a biomarker for the response to immunotherapy in various cancers [36]. Due to its stimulation by IL-1β, a pro-inflammatory cytokine, it is expected that their serum levels are correlated, which concurs with our findings regarding the pretherapeutic and follow-up serum levels. Furthermore, MIP-1α was considered a potential stimulator for the expressions of IL-1β, TNF-α, and IFN-γ due to their common effects in the macrophage-related immune response [37].

Several of the cytokines considered in our study have pro-inflammatory effects: IL-1β, IL-6, IL-8, and TNF-α. However, more importantly, these molecules were demonstrated to have involvement in tumorigenesis. IL-1β can influence the MAPK and NF-κB pathways determining various effects depending on the type of cell expressing the IL-1 receptors [38,39]. Furthermore, it was previously shown that IL-1β stimulation promoted the stemness of HNSCC cells via the Smad/ID1 pathway, while similar research on the effects of this cytokine on the tumor microenvironment revealed that IL-1β levels correlate with the stages of malignant transformation in different subtypes of HNSCC [40,41].

IL-6 is a cytokine produced in various inflammatory and immune-mediated conditions and was recently uncovered as a tumorigenic molecule in the tumor microenvironment [42,43]. Furthermore, increased expression of IL-6 was found to correlate with the development of resistance to chemotherapy in HNSCC. However, a direct link between the IL-6 pathway and the development of chemoresistance has not been proven [44].

IL-8 was shown to stimulate the release of inflammatory mediators, promoting HNSCC cell migration and increasing the expression of matrix metalloproteinases 2 and 9, known stimulating factors of the epithelial–mesenchymal transition [45,46]. HNSCC tumor progression is promoted by IL-8 via various mechanisms, such as the activation of MAPK/NF-κB and NOD1/RIP2 pathways and the reduction of JNK [46,47]. Previous studies have proposed the use of IL-8 as a biomarker in patients with treated HNSCC for predicting tumor recurrence [48].

TNF-α is a known tumor motility promoter, correlating with increased metastasis rates in OSCC patients by mediating the expression of TMEM182 and activating the ERK1/2 pathway [49]. TNF-α also promotes angiogenesis in HNSCC patients by stimulating the expression of vascular growth factors and upregulating the ERK3 pathway [50]. Along with various other cytokines, TNF-α was also considered a candidate for a biomarker in the early detection of OSCC [51], as well as in the assessment of the tumoral milieu in HNSCC, therefore playing a role in optimizing immunotherapy in these patients [52].

Among the cytokines with anti-inflammatory roles, we investigated the roles of IL-4, IL-10, and IL-6, the latter showing both pro- and anti-inflammatory effects. As we found IL-4 to be only twice as high in patients compared with controls, whereas IL-6 was 40 times higher, we can state that the possible anti-inflammatory action of IL-4 is clearly shadowed by the active pro-inflammatory action of IL-6. IL-10 was demonstrated to suppress the production of IFN-α in the HNSCC tumor microenvironment, along with other factors [53]. Moreover, serum levels of IL-10 correlate with the HNSCC stage, although its use as a biomarker for laryngeal squamous cell carcinoma has been contested in some studies [32,54]. We found IL-10 levels only five times higher in patients compared with controls; therefore, its anti-inflammatory action is probably overridden by IL-6.

IL-4 is highly expressed in the tumor microenvironment, and studies have shown that its neutralization can improve therapeutic response [55,56]. Similarly, IL-10 was shown to be upregulated in head and neck cancer and correlated with tumor progression via the JAK-STAT system, also inducing the expression of IL-6 [57]. We can state that in our investigated group, IL-6 had the highest circulatory level governing the cytokine panel and that after therapy, its level had the highest decrement, suggesting that the therapeutical protocol significantly lowered the inflammatory pattern of the patients.

The findings of this study provide an insight into the immunological mechanisms involved in OSCC pathogenesis. The cytokine profiles uncovered in our study underscore the potential for certain cytokines such as ILs 1β, 6, 8, 10, and TNF-α to be used as diagnostic tools, biomarkers of disease progression, and potential therapeutic targets. Furthermore, the cytokine profiles identified before and after treatment offer a more complex image of the pathophysiology of OSCC throughout the treatment course.

The impacts of long-term exposure to the main risk factors for OSCC—tobacco and alcohol—could influence the immunological profile, through complex mechanisms involving genetic alterations [58]. The chronic inflammation status associated with tobacco use is promoted through the activation of pro-inflammatory genes, as well as the alteration of the antitumor immune response, thus facilitating carcinogenesis [59,60]. Similar complex mechanisms that promote carcinogenesis in OSCC are reported for alcohol abuse [61]. In heavy drinkers, an increased permeability of the mucosa was detected, which promotes exposure to different carcinogens, including from tobacco, enhancing their negative impact [62]. In addition to the immunosuppression status caused by the increased production of free radicals specific to heavy drinkers, these elements can independently influence the immunological profile in both oncological and healthy subjects [63,64].

Among the limitations of our study, we can mention the relatively small sample size, caused in part by the strict inclusion criteria and specific definition of the study groups that consequently led to obtaining homogeneous and relevant patient groups. Furthermore, other types of chemokines and cytokines could reveal important roles in OSCC carcinogenesis, and future studies should include the investigation of additional molecules. Additionally, the parallel assessment of genomic alterations and gene expression for the studied cytokines would allow for stronger and more relevant correlations and is our objective for subsequent projects. Another important future perspective is the stratification of the control group based on exposure to risk factors, such as tobacco or alcohol abuse, therefore providing a more relevant comparison to the general population.

## 5. Conclusions

In this study, we have revealed that serum cytokine levels in patients with OSCC are significantly higher than in controls but are not significantly altered by treatment, therefore implying that they are also influenced by systemic factors and not only by tumor cells or by other components of the tumor microenvironment. We have also shown that the variations in the multiple cytokines correlate in the group of OSCC patients both before and after treatment, suggesting that they are regulated within specific pathways and therefore make up a serum profile governed by IL-6 that may be relevant when considering their roles as biomarkers.

Serum cytokines lie at the intersection between inflammation, the immune response, and the regulation of tumor development and progression. The complexity of the interactions between all involved cytokines and the various pathways they regulate highlights the need for further studies to clarify their definitive roles.

## Figures and Tables

**Table 1:** Comparative characteristics of subjects included in the study.

Parameter	OSCC (n = 13)	Control Group (n = 14)	*p* Value
Males (no.)	10	11	*p* = 0.7186 ^1^
Females (no.)	3	3	
Age (years ± SD)	67.92 ± 14.96	69.43 ± 11.28	*p* = 0.7692 (two-tailed) ^2^

^1^ Chi-squared test, ^2^ *t*-test (assuming equal variances).

**Table 2:** Patient characteristics.

Parameter	Number of Patients (% or Range)
Smokers	7 (53.84)
Alcohol consumption	5 (38.46)
*Tumor location*	
Lip	4 (30.76)
Oral mucosa	4 (30.76)
Tongue, pelvilingual	3 (23.07)
Gingiva	2 (15.38)
*Tumor stage (T)*	
T1	3 (23.07)
T2	6 (46.15)
T3	3 (23.07)
T4a	1 (7.69)
*Nodal stage (N)*	
N0	7 (53.84)
N1	2 (15.38)
N2	3 (23.07)
*Metastasis (M)*	
M0	13 (100)

**Table 3:** Comparative analysis of serum cytokine levels before treatment and at follow-up with the values for the control group.

Cytokine	OSCC Group		Control Group	Statistical Differences between Groups (*p*-Value)			
	Values before Treatment (n = 13)	Values at Follow-Up (n = 11)	Values (n = 14)	Three-Way Comparison ^1^	Before vs. Follow-Up^2^	Follow-Up vs. Control ^2^	Before vs. Control ^2^
MIP-1α	27.05 (1.66–321.54)	18.13 (1.67–121.53)	11.05 (1.67–25.69)	0.055	-	-	-
IL-1β	1.01 (0–2.63)	0.53 (0.14–8.84)	0.14 (0.14–0.84)	*0.006*	0.783	*0.012*	*0.003*
IL-4	0.56 (0–2.63)	0.30 (0–5.21)	0.11 (0–1.09)	0.481	-	-	-
IL-6	6.88 (1.82–919.43)	5.62 (2.01–42.49)	2.09 (0.91–4.84)	*<0.001*	0.972	*<0.001*	*<0.001*
IL-8	117.65 (7.90–1316.41)	77.33 (6.01–835.57)	12.30 (1.24–45.46)	*0.010*	0.902	*0.014*	*0.007*
IL-10	22.39 (4.77–319.07)	22.39 (6.36–57.89)	8.63 (3.18–23.22)	*0.008*	0.664	*0.019*	*0.004*
TNF-α	8.53 (3.53–109.89)	5.82 (3.29–16.50)	2.14 (1.14–6.80)	*<0.001*	0.413	*<0.001*	*<0.001*

^1^ Kruskal–Wallis test; ^2^ post hoc pairwise-comparisons test. Cytokine values are presented as median (range). Italics indicate statistically significant *p*-values.

**Table 4:** The correlation coefficient r between serum values of cytokines before treatment.

	MIP-1α	IL-1β	IL-4	IL-6	IL-8	IL-10	TNF-α
**MIP-1α**	-	0.648 *	0.409	0.544	0.949 **	0.662 *	0.949 **
**IL-1β**	-	-	0.480	0.991 **	0.659 *	0.975 **	0.408
**IL-4**	-	-	-	0.451	0.585 *	0.575 *	0.281
**IL-6**	-	-	-	-	0.562 *	0.960 **	0.289
**IL-8**	-	-	-	-	-	0.733 **	0.849 **
**IL-10**	-	-	-	-	-	-	0.405
**TNF-α**	-	-	-	-	-	-	-

* correlation with *p* values < 0.05; ** correlation with *p* values < 0.01.

**Table 5:** The correlation coefficient r between serum values of cytokines at the follow-up.

	MIP-1α	IL-1β	IL-4	IL-6	IL-8	IL-10	TNF-α
**MIP-1α**	-	0.684 *	0.815 **	0.199	0.503	0.616 *	0.858 **
**IL-1β**	-	-	0.385	0.388	0.965 **	0.918	0.438
**IL-4**	-	-	-	-0.099	0.186	0.270	0.721 *
**IL-6**	-	-	-	-	0.383	0.540	0.334
**IL-8**	-	-	-	-	-	0.885 **	0.262
**IL-10**	-	-	-	-	-	-	0.473
**TNF-α**	-	-	-	-	-	-	-

* correlation with *p* values < 0.05; ** correlation with *p* values < 0.01.

## Data Availability

The data presented in this study are available on request from the corresponding authors.

## Supplementary Materials

The following supporting information can be downloaded at: https://www.mdpi.com/article/10.3390/jcm11185398/s1, Figure S1: Serum cytokines in OSCC patients before treatment (PRE) and at follow-up (POST) and subjects in the control group (CONTROL).
