# Supplementary material for: The Assessment of Serum Cytokines in Oral Squamous Cell Carcinoma Patients: An Observational Prospective Controlled Study
Source: J Clin Med. 2022 Sep 14;11(18):5398. doi: 10.3390/jcm11185398 (PMC9503270; doi:10.3390/jcm11185398)
Supplement: Supplementary file 1 [file jcm-11-05398-s001.zip › jcm-1847839-supplementary.pdf]

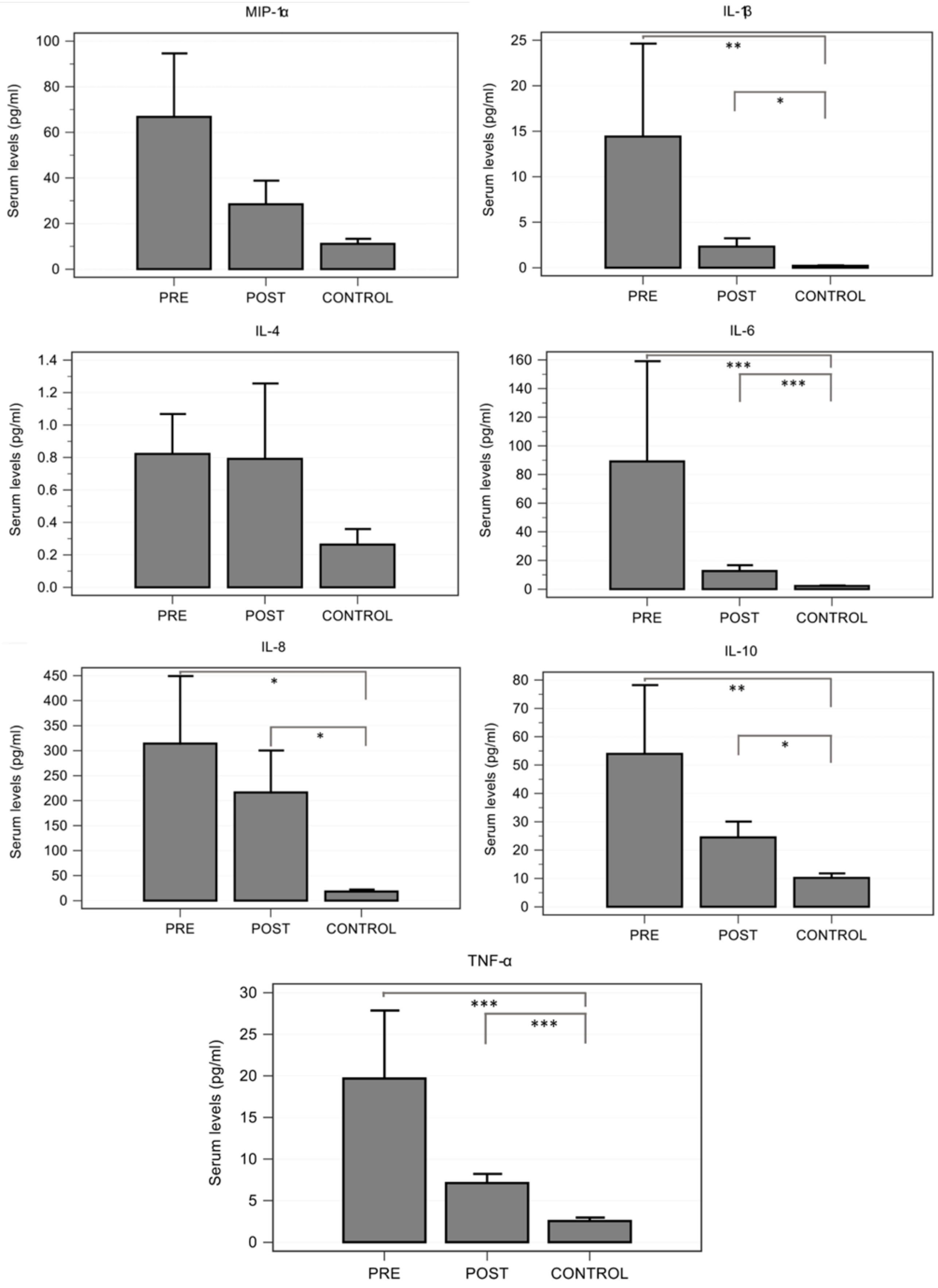

Figure S1. Serum cytokines in OSCC patients before treatment (PRE) and at follow-up (POST) and subjects in the control group (CONTROL); Error bars represent the standard error of the mean (SEM). *p <0.05, **p <0.01 and ***p <0.001.
